# Thioredoxin-Interacting Protein Promotes Phagosomal Acidification Upon Exposure to *Escherichia coli* Through Inflammasome-Mediated Caspase-1 Activation in Macrophages

**DOI:** 10.3389/fimmu.2019.02636

**Published:** 2019-11-12

**Authors:** Sung-Jin Yoon, Dong Hyun Jo, Seung-Ho Park, Jun-Young Park, Yoo-Kyung Lee, Moo-Seung Lee, Jeong-Ki Min, Haiyoung Jung, Tae-Don Kim, Suk Ran Yoon, Su Wol Chung, Jeong Hun Kim, Inpyo Choi, Young-Jun Park

**Affiliations:** ^1^Environmental Disease Research Center, Daejeon, South Korea; ^2^Fight Against Angiogenesis-Related Blindness (FARB) Laboratory, Clinical Research Institute, Seoul National University Hospital, Seoul, South Korea; ^3^Biotherapeutics Translational Research Center, Daejeon, South Korea; ^4^Immunotherapy Research Center, Korea Research Institute of Bioscience and Biotechnology (KRIBB), Daejeon, South Korea; ^5^School of Biological Sciences, College of Natural Sciences, University of Ulsan, Ulsan, South Korea; ^6^Department of Biomedical Sciences, Seoul National University College of Medicine, Seoul, South Korea

**Keywords:** thioredoxin-interacting protein, *Escherichia coli*, caspase, phagosome, macrophage

## Abstract

In host defense, it is crucial to maintain the acidity of the macrophage phagosome for effective bacterial clearance. However, the mechanisms governing phagosomal acidification upon exposure to gram-negative bacteria have not been fully elucidated. In this study, we demonstrate that in macrophages exposed to *Escherichia coli*, the thioredoxin-interacting protein (TXNIP)-associated inflammasome plays a role in pH modulation through the activated caspase-1-mediated inhibition of NADPH oxidase. While there was no difference in early-phase bacterial engulfment between *Txnip* knockout (KO) macrophages and wild-type (WT) macrophages, *Txnip* KO macrophages were less efficient at destroying intracellular bacteria in the late phase, and their phagosomes failed to undergo appropriate acidification. These phenomena were associated with reactive oxygen species production and were reversed by treatment with an NADPH oxidase inhibitor or a caspase inhibitor. In line with these results, *Txnip* KO mice were more susceptible to both intraperitoneally administered *E. coli* and sepsis induced by cecum ligation and puncture than WT mice. Taken together, this study suggests that the TXNIP-associated inflammasome-caspase-1 axis regulates NADPH oxidase to modulate the pH of the phagosome, controlling bacterial clearance by macrophages.

## Introduction

Phagocytosis by professional phagocytes, including macrophages, neutrophils, and monocytes, is a crucial process for host defense against bacterial pathogens. Phagocytosis occurs in two distinct phases, namely, bacterial internalization followed by phagosomal maturation (1). To allow for sufficient clearance of pathogenic microorganisms, phagocytes should be able to detect, engulf, and ultimately kill the pathogens (2). During phagosomal maturation, the phagosomes interact with endosomes and lysosomes, changing the phagosomal protein composition (3, 4). For example, phagosomes fuse with early endosomes and acquire the small GTPase Rab5 (5). The transition from early to late phagosomes is marked by the conversion from Rab5 to Rab7 (6). Following this, phagosomes acquire lysosomal-associated membrane protein (LAMP)-1 and LAMP-2, which are required for phagolysosomal fusion (4). The further fusion of phagosomes with lysosomes produces phagolysosomes enriched in hydrolytic enzymes, reactive oxygen species (ROS), and antimicrobial peptides, which are required for bacterial killing (4, 7).

During phagosomal maturation, the phagosomes undergo progressive acidification of the lumen, which is mainly achieved through proton pumping by the vacuolar-type H^+^-ATPase (V-ATPase) (1, 8). As phagosomes mature from early to late phagosomes, luminal pH gradually decreases from being mildly acidic (pH 6.1–6.5) to more acidic (pH 5.5–6.0) (1, 9). Furthermore, the phagolysosome is characterized by a highly acidic luminal pH (as low as 4.5) (1). In addition to its role in bactericidal activity through the generation of ROS, the activity of NADPH oxidase counteracts the activity of V-ATPase, tending toward neutralizing phagosomal pH (10–12). To ensure efficient bactericidal activity, it is therefore important to regulate the activity of proteins such as V-ATPase and NADPH oxidase in the phagosome.

One of the mechanisms responsible for regulating the activity of NADPH oxidase is the inflammasome (8). Inflammasomes are multiprotein complexes that activate caspase-1 and consist of nucleotide-binding oligomerization domain-like receptors (NLRs), such as NLR family pyrin domain containing 3 (NLRP3) and NLR family CARD domain containing 4 (NLRC4) (13). Inflammasome-activated caspase-1 causes the proteolytic processing of pro-interleukin (IL)-1β and pro-IL-18, which results in the secretion of functional IL-1β and IL-18 (14). Similarly, caspase-1 activated by the NLRP3 inflammasome accumulates in phagosomes and modulates buffering through the NADPH oxidase NOX2 to control the pH upon exposure to gram-positive bacteria (8). However, the molecular pathway regulating inflammasome-mediated NADPH oxidase has not been clearly identified.

Recent studies have shown that thioredoxin-interacting protein (TXNIP) activates the NLRP3 inflammasome under various conditions (15–18). TXNIP, first described as vitamin D3-upregulated protein 1 in acute promyelocytic leukemia HL-60 cells (19), is known to be a regulator of oxidative stress, acting as an inhibitor of the activity of thioredoxin (20, 21). In addition to these well-known functions, the dissociation of TXNIP from thioredoxin allows TXNIP to bind to NLRP3 and activate the NLRP3 inflammasome in a ROS-sensitive manner (18). Because of this, we speculated that TXNIP could be a modulator of inflammasome-mediated pH control in macrophages upon exposure to bacterial pathogens.

In this study, we investigated the roles of TXNIP in modulating the phagosomal acidity in macrophages that facilitates the destruction of bacteria through activation of the NLRP3 inflammasome-caspase-1 pathway. We first examined the clearance of bacteria by macrophages obtained from *Txnip* wild-type (WT) and knockout (KO) mice. Then, we examined the recruitment of proteins to the phagosomes, the pH of the phagosomal lumen, and the ROS levels in *Txnip* WT and KO macrophages upon treatment with bacteria. To decipher the pathways involved, specific inhibitors of the phosphoinositide 3-kinases (PI3K)/Akt pathway, V-ATPase, and caspases were employed. Based on our findings, we propose that the TXNIP-NLRP3 inflammasome-caspase-1 regulates NADPH oxidase to induce the acidification of phagosomes to clear bacteria in macrophages.

## Materials and Methods

### Animals

The animal study was approved by the Institutional Animal Care and Use Committee of the Korea Research Institute of Bioscience and Biotechnology (KRIBB-IACUC, approval number: KRIBB-AEC-11044). All procedures were performed in accordance with guidelines regarding the use of laboratory animals (National Institutes of Health). WT C57BL/6 mice were obtained from the Korea Research Institute of Bioscience and Biotechnology, and *Txnip* KO mice were prepared as previously described (16). All mice were housed in a pathogen-free animal facility under a standard light-dark cycle with standard rodent chow and water provided *ad libitum*. The experimental groups were all age- and sex-matched.

### Preparation of Peritoneal Macrophage

Mouse peritoneal macrophages were harvested and cultured as described previously (16). Cells were harvested 4 days after intraperitoneal injection of 3% thioglycollate (Sigma). Macrophages were washed and plated in a 24-well-plate at 5 × 10^5^ cells per well. After incubation with serum-free RPMI medium for 2 h at 37°C, the wells were washed three times to remove non-adherent cells, and the culture medium was replaced with RPMI supplemented with 10% fetal bovine serum (FBS) and 1% Antibiotic-Antimycotic (Thermo Fisher).

### Phagocytosis Assay

Mouse peritoneal macrophage cells were plated in 24-well-plates at 5 × 10^5^ cells per well and incubated with GFP-expressing *E. coli*, which were prepared as previously described (22). For the detection of engulfment, cells were incubated with GFP-expressing *E. coli* at indicated ratios (macrophage:bacteria CFU) at 37°C for 30 min. After the incubation, cells were washed three times with cold PBS to remove remaining bacteria, and the cells were scraped. For the detection of remaining bacteria in mouse peritoneal macrophages, cells were incubated with GFP-expressing *E. coli* at 37°C for 1 h and washed five times with cold PBS. Then, the culture medium was replaced with RPMI supplemented with 10% FBS, 1% Antibiotic-Antimycotic (Thermo Fisher), and 10 μg/ml gentamicin to inhibit the growth of extracellular bacteria for the indicated periods. Cells were analyzed immediately using a FACSCanto II flow cytometer (BD), and the data were processed using the FACSDiva software (BD). For the treatment of inhibitors, cells were incubated with 10 μM wortmannin (Selleckchem) or 20 nM bafilomycin A (Selleckchem) for 30 min before the addition of bacteria. For the phagosomal maturation assay using pHrodo™ Red *E. coli* Bioparticles, cells were plated in 48-well-plates at 2 × 10^5^ cells per well and incubated with pHrodo™ Red *E. coli* Bioparticles at 20 μg per well at the indicated periods. After the incubation, cells were washed three times with cold PBS and then immediately analyzed using a FACSCanto II flow cytometer (BD).

### Immunostaining

Cells were immunostained as previously described (22). Peritoneal macrophages (1 × 10^5^ cells per well) were plated on round glass coverslips in 24-well-plates and incubated with bacteria multiplicity of infection (MOI) of 10. For the phagocytosis of yellow-green fluorescent FluoSpheres beads of size 2.0 μm (Thermo Fisher), peritoneal macrophages were plated on round glass coverslips in 24-well-plates and incubated with 5 × 10^5^ beads/ml per well for 1 h at 37°C. After incubation, the cells were washed with cold PBS, fixed for 15 min at room temperature (RT) in 4% paraformaldehyde, and then washed again with cold PBS. Before staining with primary antibodies, cells were permeabilized for 10 min at RT in 0.2% Triton X-100 in PBS and incubated overnight at 4°C with primary antibodies specific for Lamp1 (Abcam) as indicated. Cells were then washed with PBS and incubated for 2 h at RT with Alexa Fluor 555-conjugated donkey-anti-rabbit IgG (Thermo Fisher). Nuclei were stained with 4′,6-diamidino-2-phenylindole (Thermo Fisher). The cells were imaged using a × 60 objective and an IX81 inverted microscope (Olympus). Images were obtained using the DP30BW digital camera (Olympus) and X-Cite® 120 XL light source. The acquired images were analyzed using Metamorph 7.1 program (Molecular Devices). To count the yellow-green fluorescent FluoSpheres beads, four areas of each image field of bead-containing macrophages were analyzed. For the inhibition of NADPH oxidase or caspase-1, cells were incubated with GFP-expressing bacteria for 1 h at 37°C and then incubated with 5 μM diphenyleneiodonium (DPI; Selleckchem) and 10 μM Z-VAD (Enzo) with 10 μg/ml gentamicin for 6 h at 37°C.

### Gentamicin Protection Assay

The survival of bacteria was determined with the treatment of gentamicin as previously described (23). Briefly, mouse peritoneal macrophages were incubated with *E. coli* or GFP-expressing *E. coli* for 1 h, and then the medium was replaced with the one containing 100 μg/ml gentamicin to kill extracellular bacteria. For treatment with inhibitors, the cells were incubated with 20 nM bafilomycin A (Selleckchem) for 30 min before the addition of bacteria. After 1 h, the medium was changed with the fresh one containing 10 μg/ml gentamicin at the time. The cells were washed with 1X PBS and lysed with 0.5% Triton X-100 in sterile water for 15 min at RT. Finally, the extract was plated directly onto LB agar plates and incubated at 37°C overnight.

### Isolation of Phagosomes

Phagosomes from macrophages were isolated as previously described (9). Briefly, after the incubation of peritoneal macrophages with *E. coli*, the cells were washed in cold PBS, pelleted, resuspended in 1 ml of homogenization buffer (250 mM sucrose, 3 mM imidazole, pH 7.4), and homogenized on ice using a Dounce homogenizer. Phagosomes were then isolated by flotation in a sucrose step gradient during centrifugation for 1 h at 100,000 g at 4°C. The fraction was then collected from the interface of the 10% and 25% sucrose solutions and resuspended in RIPA buffer.

### Western Blotting Analysis

For Western blotting, the protein extracts were prepared by resuspending cells or phagosomes in the lysis buffer [50 mM Tris, pH 7.5, 1 mM ethylenediaminetetraacetic acid, 150 mM NaCl, 0.1% sodium dodecyl sulfate (SDS), and 1% NP-40] containing a 1X protease inhibitor cocktail solution and 1X phosphatase inhibitor cocktail. Proteins were separated via SDS-polyacrylamide gel electrophoresis (PAGE) and subsequently transferred to Immobilon-P membranes (Millipore). Primary antibodies specific for Rab5 (catalog no. 3547), Rab7 (catalog no. 9367), caspase-1 (catalog no. 24232), and GAPDH (catalog no. 2118) were purchased from Cell Signaling. Primary antibodies specific for Lamp1 (catalog no. sc-19992) and β-actin (catalog no. sc-47778) were purchased from Santa Cruz. Primary antibodies specific for TXNIP (catalog no. K0205-3) were purchased from MBL. Primary antibodies specific for V-ATPase (catalog no. GTX110815) were purchased from GeneTex. The densitometry analysis of Western blot was carried out using Image Lab 6.0.1 (Bio-Rad).

### ROS Detection

For the detection of the amount of superoxide, mouse peritoneal macrophages were incubated with GFP-expressing *E. coli* (MOI of 20) at 37°C for 1 h, washed three times with cold PBS, and incubated with 1 μM dihydroethidium (DHE, Thermo Fisher) at 37°C for 20 min. For the demonstration of the effects of the clearance after the engulfment, cells were incubated with GFP-expressing *E. coli* (MOI of 20) at 37°C for 1 h, washed three times with cold PBS, and then incubated with 100 μg/ml gentamicin to remove the extracellular bacteria. After 1 h, the culture medium was replaced with RPMI supplemented with 10% fetal bovine serum, 1% penicillin/streptomycin, and 10 μg/ml gentamicin at the indicated periods. After 6 h of incubation of bacteria with macrophage, cells were incubated with 1 μM dihydroethidium (DHE, Thermo Fisher) at 37°C for 20 min. The GFP-positive population represented the cells engulfing bacteria. The fluorescence intensities of both GFP-positive and -negative populations were analyzed to estimate the amounts of superoxide. To measure the ROS production after 6 h of phagocytosis, mouse peritoneal macrophages were incubated with bacteria, not expressing GFP, at the indicated periods and washed three times with cold PBS. Then, cells were incubated with 2 μM H_2_DCFDA (Thermo Fisher) at 37°C for 30 min. Data were analyzed using a FACSCanto II flow cytometer (BD) and the FlowJo software (BD). For the kinetic assay of ROS production, mouse peritoneal macrophages were incubated with 1 μM dihydroethidium (DHE, Thermo Fisher) and 2 μM H_2_DCFDA (Thermo Fisher) at 37°C for 20 min, washed three times with PBS, and incubated with *E. coli*. The fluorescence was detected using SpectraMax® iD3 (Molecular Devices) and analyzed using SoftMax Pro 7.0.3 (Molecular Devices).

### pH Detection

Mouse peritoneal macrophages were plated in 24-well-plates at 5 × 10^5^ cells per well, incubated with the pHrodo™ Red *E. coli* Bioparticles (Thermo Fisher) at 37°C for 30 min, and then washed three times with the cold Live Cell Imaging Solution (Thermo Fisher). pH values were compared with a standard curve obtained by the incubation with standard pH buffers (ranging from pH 4.5–7.7, Thermo Fisher). The cells were immediately analyzed using a FACSCanto II flow cytometer (BD).

### ELISA

Mouse peritoneal macrophages were incubated with bacteria at 37°C for the indicated periods, and then the culture medium was harvested. The amounts of IL-1β were analyzed using the DuoSet ELISA (R&D). After the treatment of the TMB substrate reagent (Thermo Fisher), absorbance was measured at 450 nm using the SpectraMax iD3 Multi-Mode Microplate Reader (Molecular Devices).

### Caspase-1 Activity Assay

The activity of caspase-1 was assessed using the Caspase-1/ICE Colorimetric Protease Assay Kit (cat. no. ALX-850-211, ENZO), as recommended by the manufacturer. Briefly, mouse peritoneal macrophage protein extracts were prepared by the Cell lysis buffer contained in the Assay Kit. The protein extracts (100 μg per each sample) were incubated with 200 μM YVAD-pNA substrate at 37°C for 2 h, and the absorbance at 405 nm was read using the SpectraMax iD3 Multi-Mode Microplate Reader (Molecular Devices).

### Bacterial Infection

For bacterial infection, mice were injected intraperitoneally with live *E. coli* (5 × 10^6^ CFUs/g) at the age of 8–10 weeks (16). For the cecal ligation and puncture experiments, the cecum was ligated at half the distance between the distal pole and the base of the cecum. Cecal puncture (through-and-through) was performed from mesenteric toward antimesenteric direction after the ligation (24, 25).

### TUNEL Assay

The TUNEL assay was performed as previously described (22). Briefly, the tissues were fixed in formalin and embedded in paraffin prior to sectioning. The TUNEL assay was performed using the DeadEnd colorimetric TUNEL System (Promega), according to the manufacturer's instructions.

### Statistical Analysis

The statistical significance of differences in mean values was calculated using unpaired, two-tailed Student's *t*-test. The statistical significance of the effect for time and treatments was analyzed using two-way ANOVA. The statistical significance was determined using GraphPad software version 8.1.1 (Prism, La Jolla, CA). A *p* < 0.05 was considered statistically significant.

## Results

### TXNIP Regulates the Clearance of Bacteria From Macrophages

**Figure 1:**
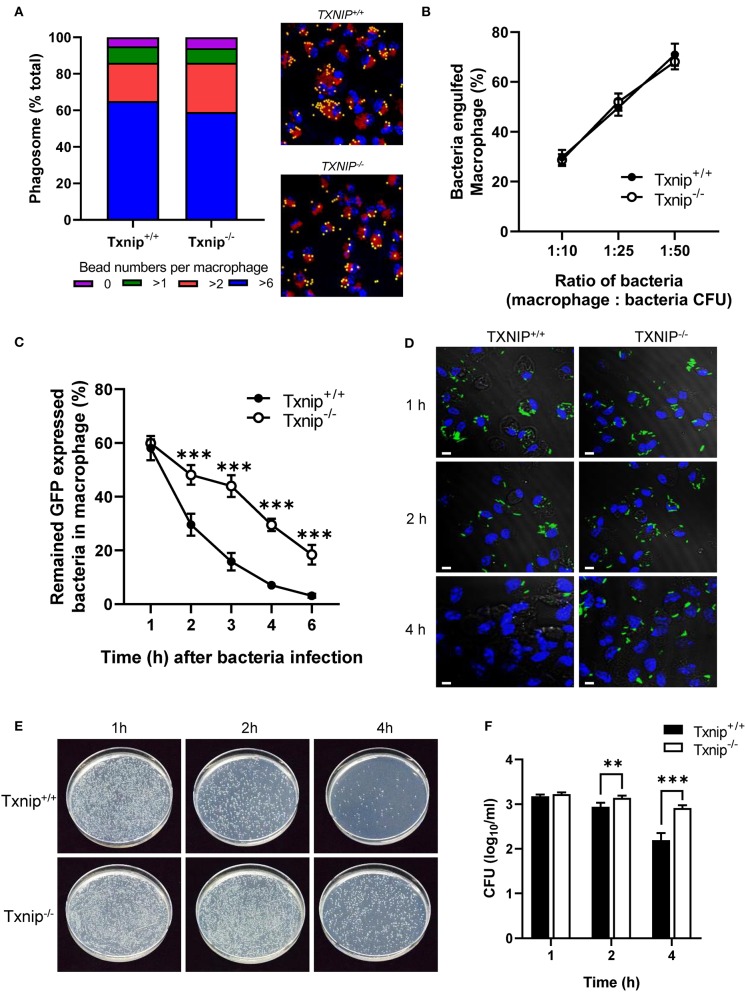
Thioredoxin-interacting protein (TXNIP) regulates the clearance of bacteria from macrophages. **(A)** (Left) The proportion of yellow-green fluorescent FluoSpheres bead numbers in wild-type (WT) and *Txnip* knockout (KO) mouse peritoneal macrophages. (Right) Representative images showing 2-μm microbeads in WT and *Txnip* KO macrophages at 1 h after incubation with microbeads (Lamp1, red; DAPI, blue). **(B)** The proportion of WT and *Txnip* KO peritoneal macrophages that engulfed GFP-expressing *E. coli* at 1 h after incubation with bacteria assessed at different macrophage to bacteria [colony-forming units (CFUs)] ratios. **(C)** The proportion of WT and *Txnip* KO peritoneal macrophages that retained GFP-expressing *E. coli* at the indicated time points after treatment with bacteria at a multiplicity of infection (MOI) of 20 (^***^P < 0.001 compared with WT). **(D)** Representative images of WT and *Txnip* KO mouse peritoneal macrophages at the indicated time points after treatment with GFP-expressing *E. coli* at an MOI of 10. Scale bars, 20 μm. **(E)** Representative LB agar plates after overnight incubation with cell extracts derived from WT and *Txnip* KO peritoneal macrophages incubated with *E. coli* for the indicated times. **(F)** CFUs on LB agar plates after overnight incubation with cell extracts derived from WT and *Txnip* KO mouse peritoneal macrophages incubated with *E. coli* for the indicated times. Data are expressed as the mean ± SD (*n* = 3, ^**^P < 0.01, ^***^P < 0.001 compared with WT).

Macrophages are professional phagocytes that engulf large particles (≥0.5 μm) including microorganisms and are capable of destroying pathogens (1). To investigate the roles of TXNIP in the regulation of phagocytosis, peritoneal macrophages were isolated from WT and *Txnip* KO mice. There was no difference in the engulfment of yellow-green fluorescent FluoSpheres beads or of GFP-expressing *E. coli* in both types of macrophages at 1 h (Figures 1A,B). To evaluate the effects of TXNIP on phagosome maturation, the fluorescence of the GFP-expressing *E. coli* in the macrophages was measured by flow cytometry and confocal microscopy at different times after bacterial uptake. To do this, after 1 h of treatment with bacteria, the medium was replaced with fresh medium to remove any extracellular bacteria. Interestingly, *Txnip* KO macrophages showed higher fluorescence levels of GFP-expressing *E. coli* than WT macrophages (Figure 1C; Supplementary Figure 1A). Furthermore, immunofluorescence microscopy also showed that *Txnip* KO macrophages retained more GFP-expressing *E. coli* than WT macrophages (Figure 1D). Based on these findings, we performed a phagocytosis assay using live imaging. In these data, we can observe that the reduction of the fluorescent bacteria is higher in the WT than in the *Txnip* KO macrophages (Supplementary Video 1). Consistent with these findings, the levels of remaining extracellular bacteria were significantly higher in *Txnip* KO macrophages than in WT macrophages (Supplementary Figure 1B). These results indicated that *Txnip* KO macrophages are less able to clear *E. coli* after engulfment compared to WT macrophages. To make sure, we assessed whether TXNIP could regulate the number of engulfed bacteria in macrophages. The gentamycin protection assay revealed that the number of bacterial cell colony-forming units (CFUs) present over time in extracts of bacterially exposed *Txnip* KO macrophages was higher than in WT macrophages (Figures 1E,F). These results indicate that TXNIP has an essential role in regulating the clearance of bacteria by macrophages.

### TXNIP Controls the Acidification of Phagosomes in Macrophages After the Engulfment of Bacteria

**Figure 2:**
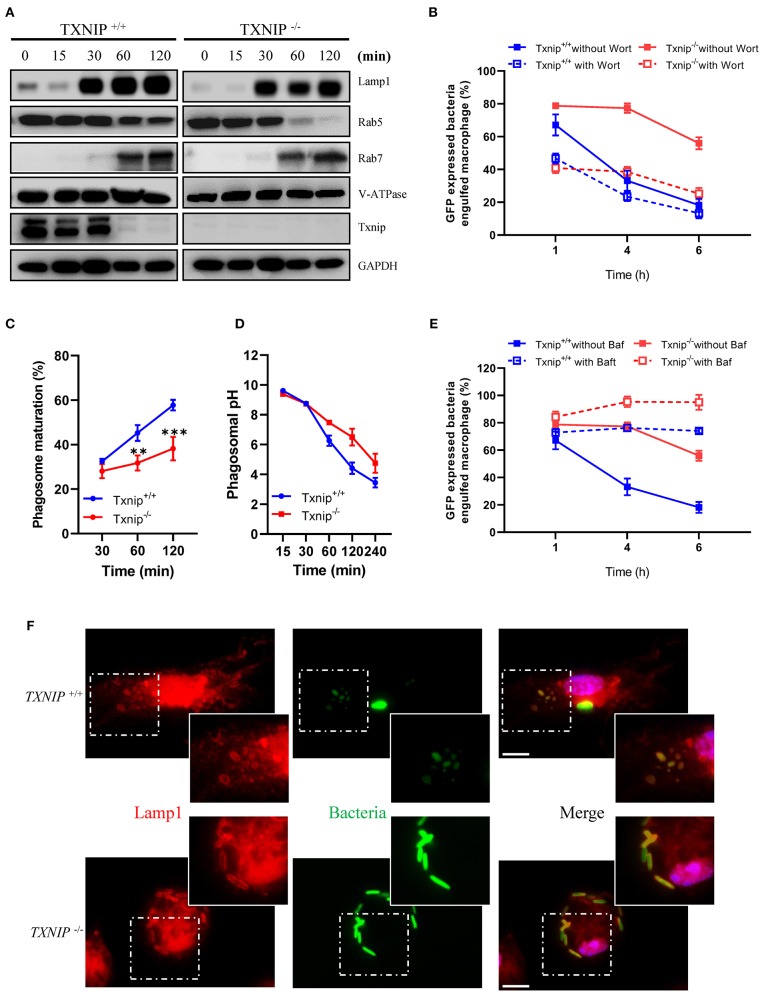
Thioredoxin-interacting protein (TXNIP) controls the acidification of phagosomes in macrophages after the engulfment of bacteria. **(A)** The expression of proteins related to phagosome maturation in phagosomes isolated from wild-type (WT) and *Txnip* knockout (KO) mouse peritoneal macrophages after treatment with *E. coli* for the indicated times. **(B)** The proportion of WT and *Txnip* KO peritoneal macrophages, treated with or without wortmannin (10 μM), which retained GFP-expressing *E. coli* after exposure to GFP-expressing *E. coli* at a multiplicity of infection (MOI) of 20 for the indicated times. (*n* = 3) **(C)** The proportion of WT and *Txnip* KO mouse peritoneal macrophages showing PE-positive phagosomes after treatment with pHrodo™ Red *E. coli* Bioparticles for the indicated times. Data are expressed as the mean ± SD (*n* = 3, ^**^P < 0.01, ^***^P < 0.001 compared with WT). **(D)** Estimated phagosomal pH in WT and *Txnip* KO mouse peritoneal macrophages after treatment with pHrodo™ Red *E. coli* Bioparticles for the indicated times. **(E)** The proportion of WT and *Txnip* KO mouse peritoneal macrophages, treated with bafilomycin A (20 nM), which retained GFP-expressing *E. coli* after incubation with GFP-expressing *E. coli* at an MOI of 20 for the indicated times (*n* = 3). **(F)** Representative images of bacteria-laden WT and *Txnip* KO mouse macrophages 6 h after a 1-h treatment with GFP-expressing *E. coli* and removal of extracellular bacteria. Scale bar, 10 μm.

Rab5 and Rab7 are markers for early and late phagosomes, respectively, whereas LAMP-1 and V-ATPase are markers for phagolysosome fusion (4, 26, 27). Although *Txnip* KO macrophages showed a decreased clearance of bacteria after engulfment, there were no differences in the protein expression levels of Rab7, LAMP-1, and V-ATPase in phagosomes isolated from WT and *Txnip* KO macrophages after treatment with bacteria. However, *Txnip* KO macrophages recruited less Rab5 into the phagosomes at 1 and 2 h after the treatment of bacteria (Figure 2A; Supplementary Figure 2), although there were no differences in total Rab5 expression in whole-cell lysates of *Txnip* WT and KO macrophages (Supplementary Figures 3A,B). These results suggest that TXNIP might be involved in the recruitment of Rab5 to the phagosomes in the early phase (1–2 h after the treatment of bacteria). To investigate whether these effects on differential Rab5 expression were related to the clearance of the bacteria in the late phase (4–6 h after infection with bacteria as shown in Figures 1C–F; Supplementary Figures 1A,B), we pretreated macrophages with wortmannin (Supplementary Figure 3C), an inhibitor of the PI3K/Akt pathway, which modulates Rab5 recruitment to phagosomes (26). Interestingly, wortmannin decreased the engulfment of bacteria in both *Txnip* WT and KO macrophages but did not affect the different patterns of bacterial clearance between both cell types (Figure 2B).

As stated above, WT and *Txnip* KO macrophages showed similar patterns of recruitment of Rab7, LAMP-1, and V-ATPase to the phagosomes (Figure 2A). On the other hand, we found that there was a striking difference in phagosome maturation between WT and *Txnip* KO macrophages in terms of acidification (Figures 2C,D). To assess phagosomal maturation, macrophages were incubated with pHrodo Red *E. coli* Bioparticles, which is an indicator for phagolysosomal maturation, for the indicated times. The data showed that WT macrophages had higher levels of phagolysosomal maturation compared with *Txnip* KO macrophages (Figure 2C). The pH, which indicates acidification in the phagosomes, was also significantly higher in phagosomes from *Txnip* KO macrophages than in phagosomes from WT macrophages (Figure 2D). V-ATPases are known to play essential roles in the acidification of phagosomes (27). As shown in Figure 1C, we assessed whether V-ATPase activity inhibition could reduce the number of engulfed bacteria in WT macrophages compared to that in *Txnip* KO macrophages. When WT macrophages were pretreated with bafilomycin A (Supplementary Figure 3C), an inhibitor of V-ATPase (28, 29), they showed dysfunction in bacteria clearance with a definite defect in destroying, but not reducing the number of engulfed GFP-expressing *E. coli* (Figure 2E; Supplementary Figures 4A,B). Similar to that in WT macrophages, V-ATPase inhibition also totally prevents the bacteria clearance upon phagocytosis in *Txnip* KO macrophages (Figure 2E; Supplementary Figures 4A,B). Our observation suggests that V-ATPase inhibition halts the clearance of engulfed bacteria and not only reduced it. So, the data suggest that pathways regulating the acidification of phagosomes other than V-ATPase might be impaired in these cells. Nevertheless, an immunofluorescence analysis for the staining of Lamp1, known as a late phagosomal marker, showed that the bacteria are more fluorescent in the *Txnip* KO macrophages. In contrast, it is difficult to distinguish their shape in the WT macrophage (Figure 2F). These data suggest that TXNIP can regulate *E. coli* survival in the phagosomes by lowering the phagosomal pH.

### TXNIP Regulates the Level of Superoxide in Macrophages After the Engulfment of Bacteria

**Figure 3:**
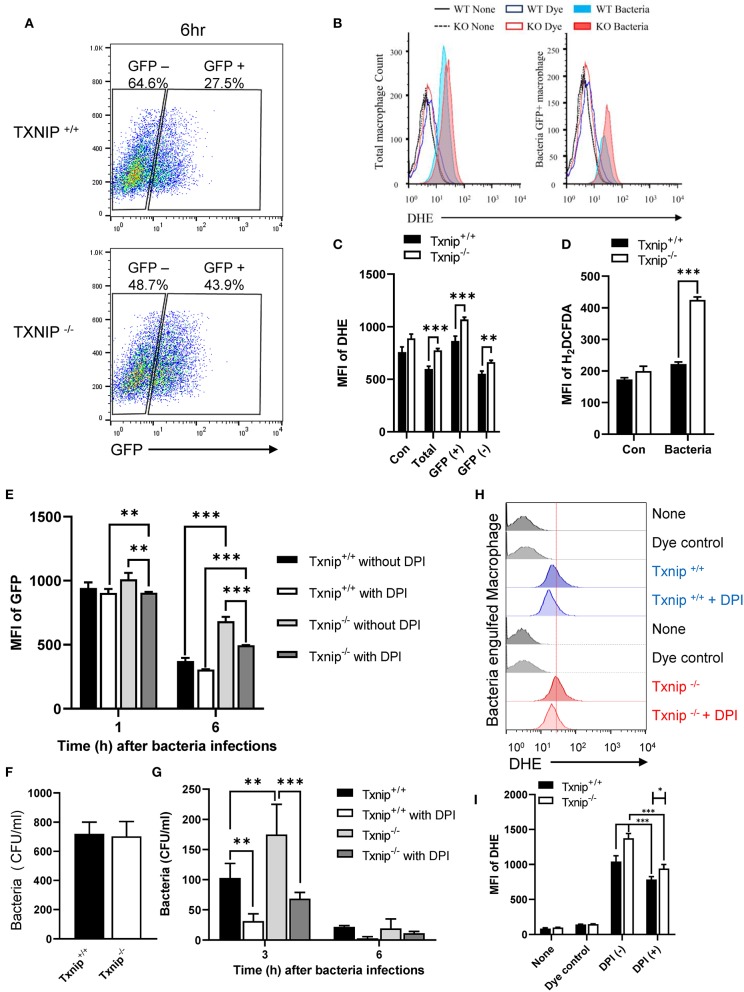
Thioredoxin-interacting protein (TXNIP) regulates the level of superoxide in macrophages after the engulfment of *E. coli*. **(A)** FACS analyses showing the proportion of wild-type (WT) and *Txnip* knockout (KO) mouse peritoneal macrophages that retained GFP-expressing *E. coli* 6 h after treatment with GFP-expressing *E. coli* at a multiplicity of infection (MOI) of 20. **(B)** The distribution of WT and *Txnip* KO mouse peritoneal macrophages based on the intensity of the DHE dye after treatment with GFP-expressing *E. coli* at an MOI of 20 at 6 h. **(C)** The mean fluorescence intensity (MFI) of the DHE dye in the total, GFP-positive, and GFP-negative WT and *Txnip* KO mouse peritoneal macrophages after treatment with GFP-expressing *E. coli* at an MOI of 20 at 6 h. Data are expressed as the mean ± SD (*n* = 3, ^**^P < 0.01, ^***^P < 0.001 compared with WT). **(D)** The MFI of the H_2_DCFDA dye in WT and *Txnip* KO mouse peritoneal macrophages after treatment with *E. coli* at an MOI of 20 at 6 h. Data are expressed as the mean ± SD (*n* = 3, ^***^P < 0.001 compared with WT) **(E)** The MFI of the GFP in WT and *Txnip* KO mouse macrophages at 1 and 6 h after treatment with GFP-expressing *E. coli* at an MOI of 20 with or without DPI treatment. Data are expressed as the mean ± SD (*n* = 3, ^**^P < 0.01, ^***^P < 0.001 compared with WT) **(F)** CFUs on LB agar plates after overnight incubation with cell extracts derived from WT and *Txnip* KO mouse peritoneal macrophages treated with *E. coli* for 1 h at an MOI of 20 (*n* = 3). **(G)** Colony-forming units (CFUs) on LB agar plates after overnight incubation with cell extracts derived from WT and *Txnip* KO mouse peritoneal macrophages treated with *E. coli* at an MOI of 20 for the indicated times, with or without DPI treatment. Data are expressed as the mean ± SD (*n* = 3, ^**^P < 0.01, ^***^P < 0.001 compared with WT). **(H)** The distribution of WT and *Txnip* KO mouse peritoneal macrophages based on the intensity of the DHE dye at 6 h after the 1-h treatment with GFP-expressing *E. coli* at an MOI of 20, with or without DPI treatment, and removal of extracellular bacteria. (**I**) The MFI of DHE for (**H**). Data are expressed as mean (*n* = 3, ^*^P < 0.05, ^***^P < 0.001 compared with WT).

ROS produced by NADPH oxidase causes active and stable alkalization of the phagosomal lumen (10, 12), counteracting the activity of V-ATPase (8). Because the total amount of ROS produced during phagocytosis immediately increased upon bacterial infection, we assessed the kinetics of ROS production in WT and *Txnip* KO macrophages upon phagocytosis of bacteria during 1 h 30 min. After 30 min of treatment with ROS-detection dye, peritoneal macrophages were incubated with *E. coli*, and then the ROS level was measured at the indicated times. The results of kinetics showed a similar increase in ROS production between WT and *Txnip* KO macrophages (Supplementary Figures 5A,B). Thus, we conclude that TXNIP is not relevant for ROS production in the initial stage of phagocytosis. Next, we conducted a FACS analysis to determine whether the level of superoxides was affected by TXNIP and analyzed the levels of superoxides in WT and *Txnip* KO macrophages after the internalization of *E. coli*. After 1 h of treatment with GFP-expressing *E. coli*, we measured ROS levels in WT and *Txnip* KO macrophages. As in earlier experiments, there was no difference in the proportion of GFP-positive cells in WT and *Txnip* KO cells (Supplementary Figure 5C). Upon infection with bacteria, ROS levels increased in both WT and *Txnip* KO macrophages in terms of the mean fluorescence intensities (MFIs) of dihydroethidium (DHE) and total H_2_DCFDA dyes (Supplementary Figures 5D–F). It is noteworthy that GFP-negative cells showed similar intensities in DHE compared to control cells. In contrast, the proportion of bacteria-laden macrophages was higher in *Txnip* KO macrophages than in WT macrophages 6 h after infection with bacteria (Figure 3A). Also, we have measured the ROS production after 6 h of phagocytosis. The production of ROS increased to higher levels in *Txnip* KO macrophages than in WT macrophages at 6 h (Figures 3B–D). Although we did not continuously measure the level of total ROS from initiation of phagocytosis to late time points, our results showed that the level of ROS production increased more in *Txnip* KO macrophages at the late time. Thus, these results indicated that TXNIP might regulate NADPH oxidase-induced superoxide production following infection of macrophages by *E. coli* upon phagocytosis at the late time.

To investigate the effects of NADPH oxidase on the removal of bacteria, we treated macrophages with DPI to inhibit NADPH oxidase (Supplementary Figure 6A). DPI treatment decreased the proportion of *Txnip* KO macrophages containing GFP-expressing bacteria (Figure 3E). There were no differences in the CFUs present in cell extracts from WT and *Txnip* KO macrophages after 1 h of bacteria treatment (Figure 3F). On the other hand, the CFUs present in cell extracts from *Txnip* KO cells were higher than those in WT cells at 3 and 6 h after infection with bacteria (Figure 3G). Interestingly, the CFUs present in both the WT and *Txnip* KO cell extracts were decreased by DPI treatment. In addition, there was a change in bacterial shape in the phagosomes of DPI-treated *Txnip* KO macrophages, indicative of bacterial degradation (Supplementary Figure 6B). In line with these results, DPI treatment after 2 h of phagocytosis reduced the level of DHE dye in both WT and *Txnip* KO cells after bacterial treatment (Figures 3H,I). These data indicate that decreasing superoxide levels by inhibiting NADPH oxidase at late stages of phagocytosis restores the clearance of engulfed bacteria in *Txnip* KO macrophages.

### The TXNIP-NLRP3 Inflammasome-Caspase-1 Axis Regulates the Clearance of Bacteria From Macrophages

**Figure 4:**
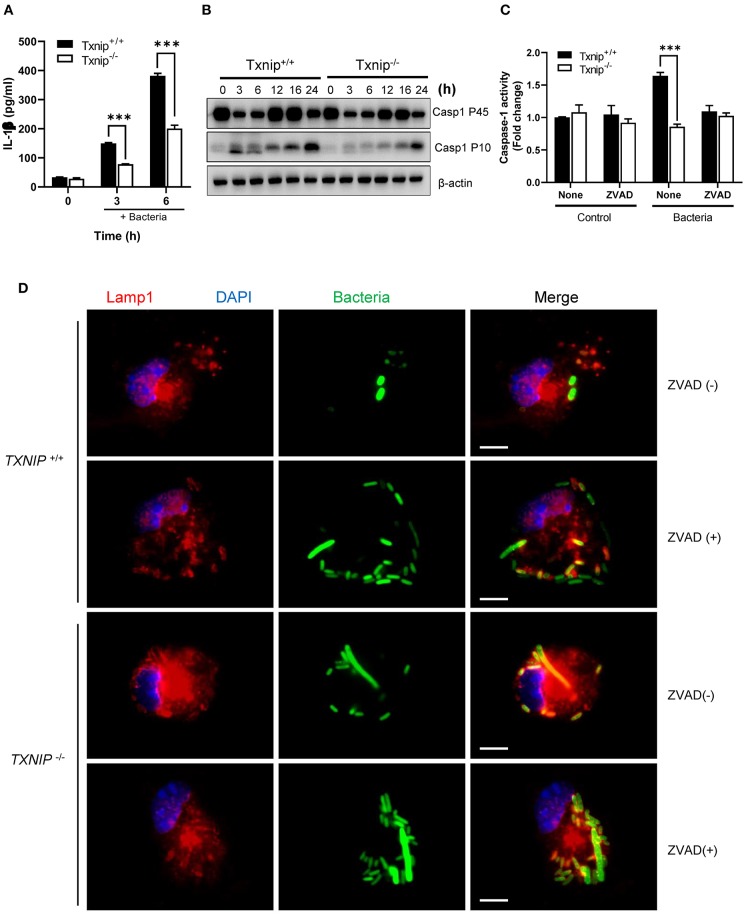
The thioredoxin-interacting protein (TXNIP)-inflammasome-caspase-1 axis regulates the clearance of bacteria from macrophages. **(A)** The levels of interleukin (IL)-1β in conditioned media from wild-type (WT) and *Txnip* knockout (KO) mouse peritoneal macrophages after treatment with *E. coli* for the indicated times. Data are expressed as the mean ± SD (^***^P < 0.001 compared with WT). **(B)** The protein expression levels of caspase-1 P45 and caspase-1 P10 in mouse WT and *Txnip* KO mouse peritoneal macrophages after treatment with *E. coli* for the indicated times. **(C)** Caspase-1 activity, with or without ZVAD treatment, in mouse WT and *Txnip* KO mouse peritoneal macrophages 6 h after the treatment with *E. coli*. Data are expressed as the mean ± SD (^***^P < 0.001 compared with WT). **(D)** Representative images of bacteria-laden WT and *Txnip* KO mouse macrophages 6 h after a 1-h treatment with GFP-expressing *E. coli* with or without ZVAD treatment and removal of extracellular bacteria. Scale bar, 10 μm.

Having shown that TXNIP regulates ROS productions by controlling NADPH oxidase, we next tried to determine the mechanism by which TXNIP controls the NLPR3 inflammasome. The interaction between TXNIP and NLRP3 causes caspase-1 activation and IL-1β secretion, and the lack of TXNIP inhibits the formation of the NLRP3-ASC-caspase-1 complex by nitrosylation (15, 16, 18, 30). Therefore, TXNIP is crucial to control the activity of the NLRP3-ASC-caspase-1 complex both directly and indirectly. During bacterial infection, it has been demonstrated that the activation of caspase-1 by NLRP3 inflammasome inhibits the action of NADPH oxidase (8). We assessed whether TXNIP could regulate the activity of NLPR3 inflammasomes following bacterial infection. Treatment with *E. coli* increased IL-1β secretion in both WT and *Txnip* KO cells, but the magnitude was lesser in *Txnip* KO cells than in WT cells (Figure 4A). Similarly, *Txnip* KO macrophages expressed lower levels of the activated form of caspase-1 (Casp1 p10) than WT macrophages (Figure 4B; Supplementary Figure 7). To assess the contribution of caspase-1 in phagosome maturation, we used the caspase-1 inhibitor, ZVAD. Following bacterial infection, caspase-1 activity increased in WT macrophages but not in *Txnip* KO macrophages. This increased caspase-1 activity seen in WT macrophages was completely ablated by ZVAD treatment (Figure 4C). Under the same conditions, ZVAD-treated WT macrophages retained the same level of intact GFP-expressing bacteria as *Txnip* KO macrophages (Figure 4D). Together, these data suggest that TXNIP improves phagosome maturation by the activation of caspase-1 through regulation of the inflammasome.

### Bacterial Clearance Is Reduced in *Txnip* KO Mice

**Figure 5:**
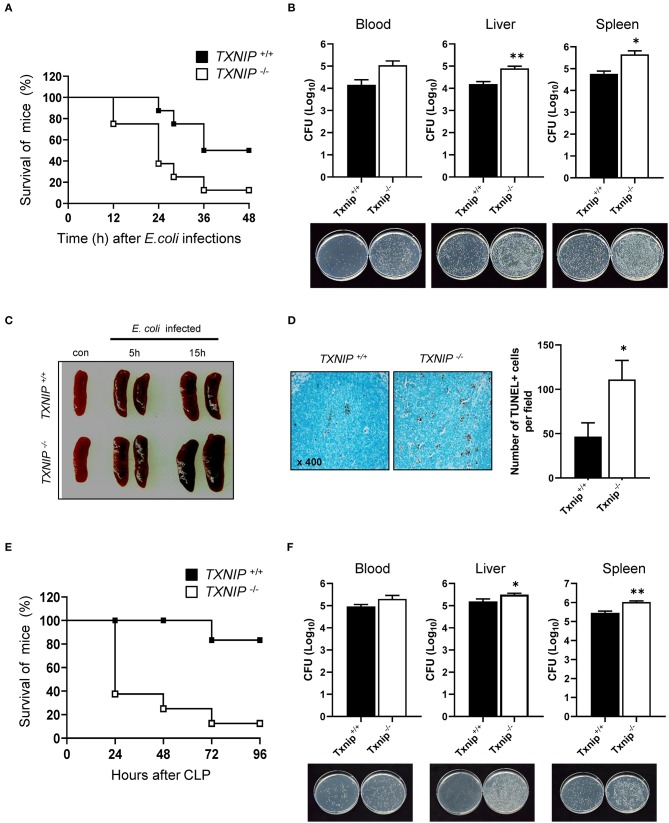
Bacterial clearance is reduced in thioredoxin-interacting protein (*Txnip*) knockout (KO) mice. **(A)** Survival of wild-type (WT) and *Txnip* KO mice after intraperitoneal injection with *E. coli* (10^8^ CFU, mice *n* = 10). **(B)** Colony-forming units (CFUs) and representative images of LB agar plates after overnight incubation with extracts of blood, liver, and spleen from WT and *Txnip* KO mice obtained 24 h after the intraperitoneal injection of *E. coli*. Data are expressed as the mean ± SD (*n* = 3, ^*^P < 0.05, ^**^P < 0.01 compared with WT). **(C)** Representative images of spleens from WT and *Txnip* KO mice at the indicated times after the intraperitoneal injection of *E. coli* (10^8^ CFU). **(D)** (Left) TUNEL-positive cells in liver sections from WT and *Txnip* KO mice 24 h after the intraperitoneal injection of *E. coli* (10^8^ CFU). (Right) The number of TUNEL-positive cells in five randomly selected fields in liver sections from WT and *Txnip* KO mice 24 h after the intraperitoneal injection of *E. coli* (10^8^ CFU). Data are expressed as the mean ± SD (^*^P < 0.05 compared with WT). **(E)** Survival of WT and *Txnip* KO mice after cecum ligation and puncture (*n* = 7). **(F)** CFUs and representative images of LB agar plates after overnight incubation with extracts of blood, liver, and spleen from WT and *Txnip* KO mice obtained 24 h after cecum ligation and puncture. Data are expressed as the mean ± SD (*n* = 3, ^*^P < 0.05, ^**^P < 0.01 compared with WT).

To confirm that the delay in bacterial clearance caused by TXNIP loss contributed to the death of mice, we performed an intraperitoneal injection of *E. coli* (10^8^ CFU) in WT and *Txnip* KO mice. *Txnip* KO mice were more susceptible to intraperitoneally administered *E. coli* than WT mice (Figure 5A). The CFUs present in the blood, liver, and spleen of WT and *Txnip* KO mice challenged with bacteria were markedly higher in *Txnip* KO mice than in the same organs from WT mice (Figure 5B). In addition, in the *Txnip* KO mice, splenomegaly was more evident (Figure 5C), and the level of apoptosis in the liver parenchyma was higher (Figure 5D). We further assessed the role of TXNIP on mouse survival in other models of bacterial challenge. The cecum ligation and puncture (CLP) model is the most widely utilized model of sepsis (24, 25). Similar to the intraperitoneal injection model, *Txnip* KO mice were more susceptible to CLP (Figure 5E), displaying a similar mortality pattern. In addition, the CFUs present in the blood and extracts from liver and spleen were also higher in *Txnip* KO mice (Figure 5F). These data suggest that TXNIP has an essential role in the defense mechanism used by animals to combat bacterial infection.

**Figure 6:**
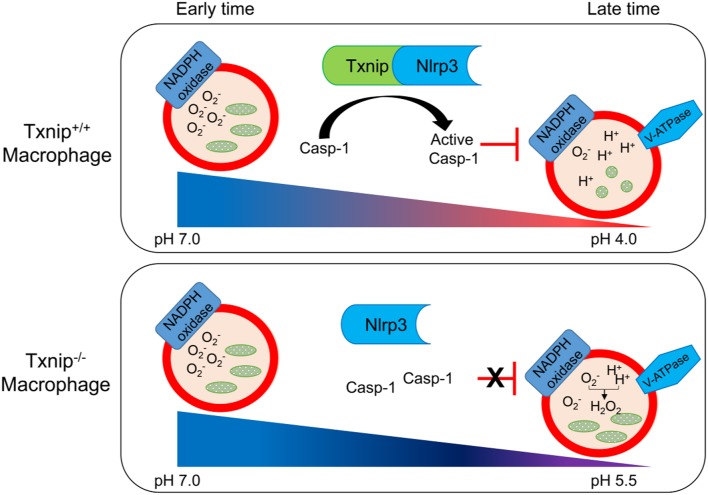
Schematic diagram showing the roles of thioredoxin-interacting protein (TXNIP)-inflammasome-caspase-1 in the clearance of bacteria from macrophages. Wild-type (WT) and *Txnip* knockout (KO) mouse macrophages demonstrate a similar engulfment of bacteria at the early stage of phagosome maturation. There is no difference in the levels of superoxide and the pH in phagosomes from WT and *Txnip* KO mouse macrophages. On the other hand, at the late stage of phagosome maturation, binding between TXNIP and NLRP3 induces the inflammasome complex to activate caspase-1, inhibiting the activity of NADPH oxidase, leading to a decrease in the pH of the phagosomal lumen. At this stage, there are significant differences in the levels of superoxide, the pH of the phagosomes, and the resultant clearance of bacteria in *Txnip* WT and KO macrophages.

Based on these findings, we propose a schematic model to describe the role of TXNIP in the phagosome maturation of macrophages (Figure 6). In this model, the TXNIP-inflammasome-caspase-1 axis regulates NADPH oxidase to modulate the pH of the phagosome, deciding the clearance of bacteria from macrophages.

## Discussion

In this study, we demonstrated that the TXNIP-NLRP3 inflammasome-caspase-1 pathway regulates NADPH oxidase to affect the acidification of the phagosomes in macrophages (Figure 6). *Txnip* KO macrophages showed less secretion of IL-1β and activation of caspase-1 upon treatment with bacteria. This might lead to disinhibition of NADPH oxidase by activated caspase-1, resulting in increased ROS levels and pH. Accordingly, *Txnip* KO macrophages fail to clear bacteria adequately.

*Txnip* KO macrophages showed a clear defect in the clearance of engulfed *E. coli*, but not in engulfment itself. Accordingly, we investigated the effects of TXNIP depletion on several aspects of phagosome maturation, including the recruitment of phagosomal proteins, the phagosomal pH, and bacterial degradation. Even though clearance during the late phase was affected, there were no differences in the recruitment of Rab7, LAMP-1, and V-ATPase to the phagosomes. Instead, *Txnip* KO macrophages demonstrated clear changes in pH, ROS production, the secretion of IL-1β, and the activation of caspase-1.

It is known that ROS generated by NOX2 is important for removing bacteria in the phagosome, forming LAP (LC3 associated phagosome), a type of phagosome created by LC3, and presenting the antigen (31–34). Although NOX2 has microbicidal activity, the formation of superoxide by NOX2 can inhibit phagosomal acidification by V-ATPase, and the cellular damage caused by sustained ROS can induce apoptosis (8, 35, 36). The removal of bacteria by NOX2 and the formation of LAP are important mechanisms in the early stages of macrophage contact with bacteria, but if the activity of this enzyme is not continuously controlled in the phagosome at later times, it will affect the viability of the bacteria in the phagosome. In commensal bacteria, the low level of ROS generated by NADPH oxidase modulates the redox-sensor regulatory signaling pathway and supports symbiotic effects of the bacteria (37). In this context, the NADPH oxidase-dependent generation of ROS has a double-edged sword effect in multicellular organisms and is connected to the adaptation or survival of the microbiome.

Acidification is the key to the many facets of phagosome maturation. It is a tightly regulated process that begins after the phagocytic cup has closed and phagosome luminal pH goes from 7 to 4. These changes precede the fusion with acidic compartments, and this early acidification event requires delivery of the V-ATPase (17). This proton-transporting enzyme is recruited from endosomes and lysosomes and is assembled on the membrane of the nascent vacuole (15). However, how the pH is then regulated remains poorly defined for the phagosomal state. In early phases following phagocytosis of gram-positive bacteria, there is rapid acidification of the phagosomal lumen, and the NLRP3-dependent inflammasome complex generates activated caspase-1, which regulates the superoxide generated by NOX2. In this early process, it has been shown that gram-negative bacteria cannot trigger this mechanism (8). However, our results suggest that TXNIP modulates the levels of superoxide to regulate the activity of caspase-1, generated by the NLRP3 inflammasome complex at later stages, and induces bactericidal activity toward engulfed *E. coli* by acidifying the phagosomal lumen in order to activate acidic enzymes present in the phagosome. Although the bacteria clearance of *Txnip* KO macrophages incubated with DPI did not completely restore to the level of that in WT macrophages, the survival of engulfed bacteria in *Txnip* KO macrophages was reduced compared with that of KO macrophages incubated without DPI at the late time (Figure 3). Our result of isolated phagosomes showed that Rab5 is less recruited into the phagosomes of *Txnip* KO macrophages, and the reduced level of Rab5 did not influence the acidification of phagosomal lumen at the late time. Commonly, Rab5 is known as a regulator of an early endosome or phagosome formation, and this protein is needed in phagosome maturation for acidification of the phagosomal lumen and is essential to reduce bacterial evasion from the host cells (1, 5). In this context, our results indicated the possibility that TXNIP can regulate the formation of Rab5-dependent phagosomes and the acidification against invading bacteria in the early time. As a result, the difference of Rab5 level into the phagosome may be influenced by the bacterial clearance in the *Txnip* KO macrophage incubated with DPI (Figure 2A). To evade these processes, some pathogens have evolved mechanisms such as buffering their local environment in an attempt to maintain a beneficial neutral pH. For this reason, strict regulation of phagosomal maturation is crucial for bactericidal activity in macrophages and to regulate the immune system.

Gram-negative bacteria are associated with pneumonia, bloodstream infections, and urinary tract infections, and these bacteria can easily acquire antibiotic drug resistance genes (38). Macrophages are an important part of the inflammatory response to these bacteria, modulating the activity of the immune system via the production of cytokines and chemokines and providing clearance through their phagocytic machinery (2, 39, 40). In a sophisticated phagocytosis system to protect against invading bacteria, the survival of *E. coli* in the phagosome machinery is essential for it to adapt to the host or else a mutation that results in the acquisition of antibiotic resistance is required (37, 41–43). In order to survive within macrophages, bacteria have developed strategies to escape the phagocytic machinery using bacterial effector protein, actin modulation, and alkalization of the phagosomal lumen (1, 22, 44, 45). In this context, complete phagosomal maturation is crucial to destroy bacteria and to regulate the host immune system. Our results demonstrate that the regulation of NADPH oxidase-derived superoxide via the TXNIP-NLRP3 inflammasome-caspase-1 axis induces a continuous acidification of the phagosomal lumen that inhibits bacterial survival.

Recently, numerous studies have shown that the cytosolic levels of TXNIP are important in regulating diversity cellular signaling pathways, such as the redox system, inflammation, glucose uptake, and apoptosis (17, 30, 46–48). In the same manner, differential TXNIP expression levels are thought to be important in regulating cellular or tissue homeostasis during bacterial infection. Our results show that the TXNIP-NLRP3 inflammasome-caspase-1 axis is a modulator of the phagosomal pH in macrophages exposed to bacteria. Without this effective system, macrophages will fail to clear *E. coli* even in the presence of increased ROS levels. We suggest that TXNIP might play a role in the destruction of pathogenic *E. coli* through the effects of inflammasome-mediated caspase-1 on NADPH oxidase.

## Data Availability Statement

The raw data supporting the conclusions of this manuscript will be made available by the authors, without undue reservation, to any qualified researcher.

## Ethics Statement

The animal study was reviewed and approved by The animal study was approved by Institutional Animal Care and Use Committee of the Korea Research Institute of Bioscience and Biotechnology (KRIBB-IACUC, approval number: KRIBB-AEC-11044) and all procedures were performed in accordance with guidelines regarding the use of laboratory animals (National Institutes of Health). Written informed consent was obtained from the owners for the participation of their animals in this study.

## Author Contributions

S-JY, DJ, and S-HP performed experiments, data analysis, contributed to the study design, and drafting of the manuscript. J-YP and Y-KL assisted with some experiments and data analysis. M-SL, J-KM, SC, and JK were responsible for setting up the experiments, interpretation of data, and critically reviewing the manuscript. HJ, T-DK, and SY provided useful suggestions. IC and Y-JP supervised the study, collected the data, and wrote the manuscript.

### Conflict of Interest

The authors declare that the research was conducted in the absence of any commercial or financial relationships that could be construed as a potential conflict of interest.

## Supplementary Material

Supplementary Video 1 **(Left)** Txnip WT and **(Right)** Txnip KO mouse peritoneal macrophages were infected with GFP-expressing *E. coli*. Phagocytosis was initiated approximately 5 min after cells were exposed to bacteria.
