# Supplementary material for: Thioredoxin-Interacting Protein Promotes Phagosomal Acidification Upon Exposure to Escherichia coli Through Inflammasome-Mediated Caspase-1 Activation in Macrophages
Source: Front Immunol. 2019 Nov 12;10:2636. doi: 10.3389/fimmu.2019.02636 (PMC6861186; doi:10.3389/fimmu.2019.02636)
Supplement: Supplementary file 2 [file Data_Sheet_1.pdf]

Supplementary Material

Supplementary Figure

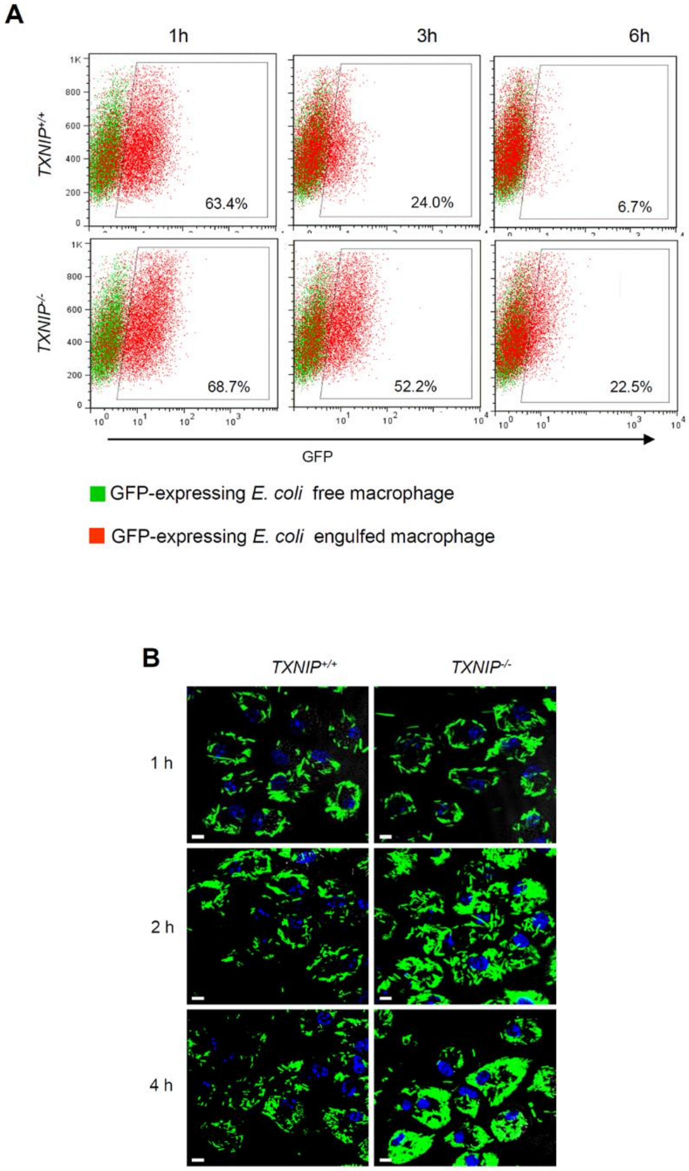

Supplementary Figure 1. Delay in the clearance of bacteria by *Txnip* KO macrophages. (A) FACS analysis showing the proportion of WT and *Txnip* KO mouse peritoneal macrophages, which retained GFP-expressing *E. coli* at the indicated time points after treatment with bacteria at an MOI of 10. (B) Representative images of WT and *Txnip* KO mouse peritoneal macrophages at the indicated time points after treatment with GFP-expressing *E. coli* at an MOI of 50, without removal of extracellular bacteria. Scale bars, 20 μm.

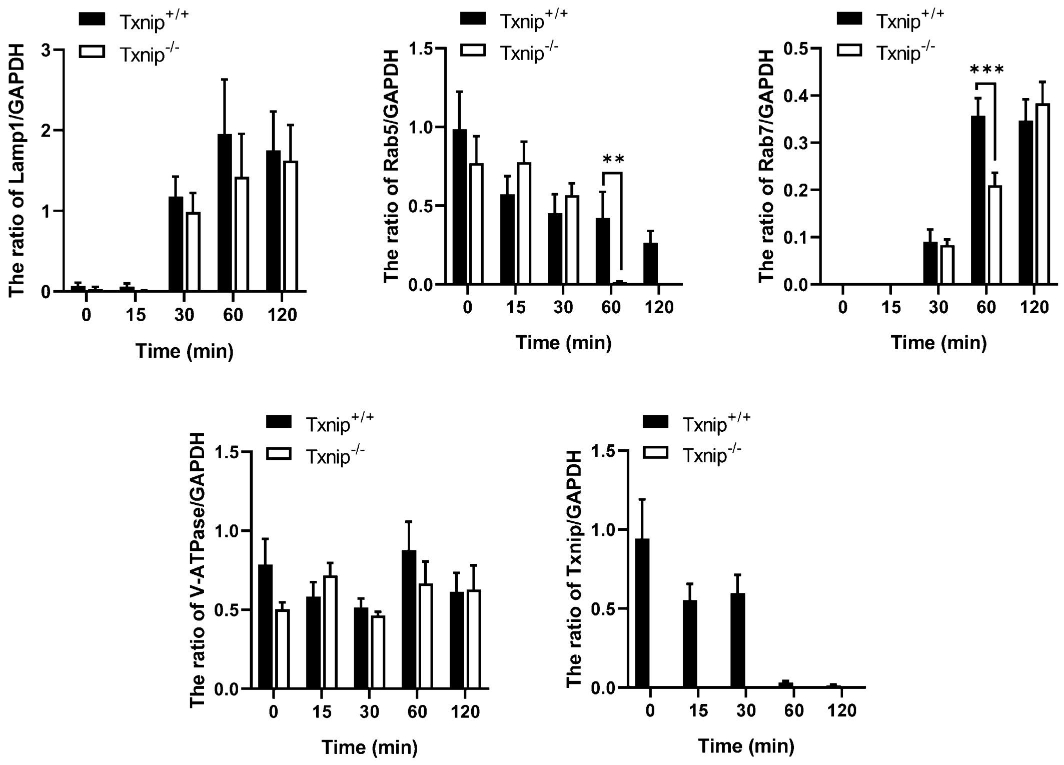

Supplementary Figure 2. Quantification data of Lamp1, Rab5, Rab7, V-ATPase, and Txnip determined using western blotting data presented in Figure 2A. Data are expressed as mean ± SD (n = 3, **P < 0.01, ***P < 0.001, compared with WT).

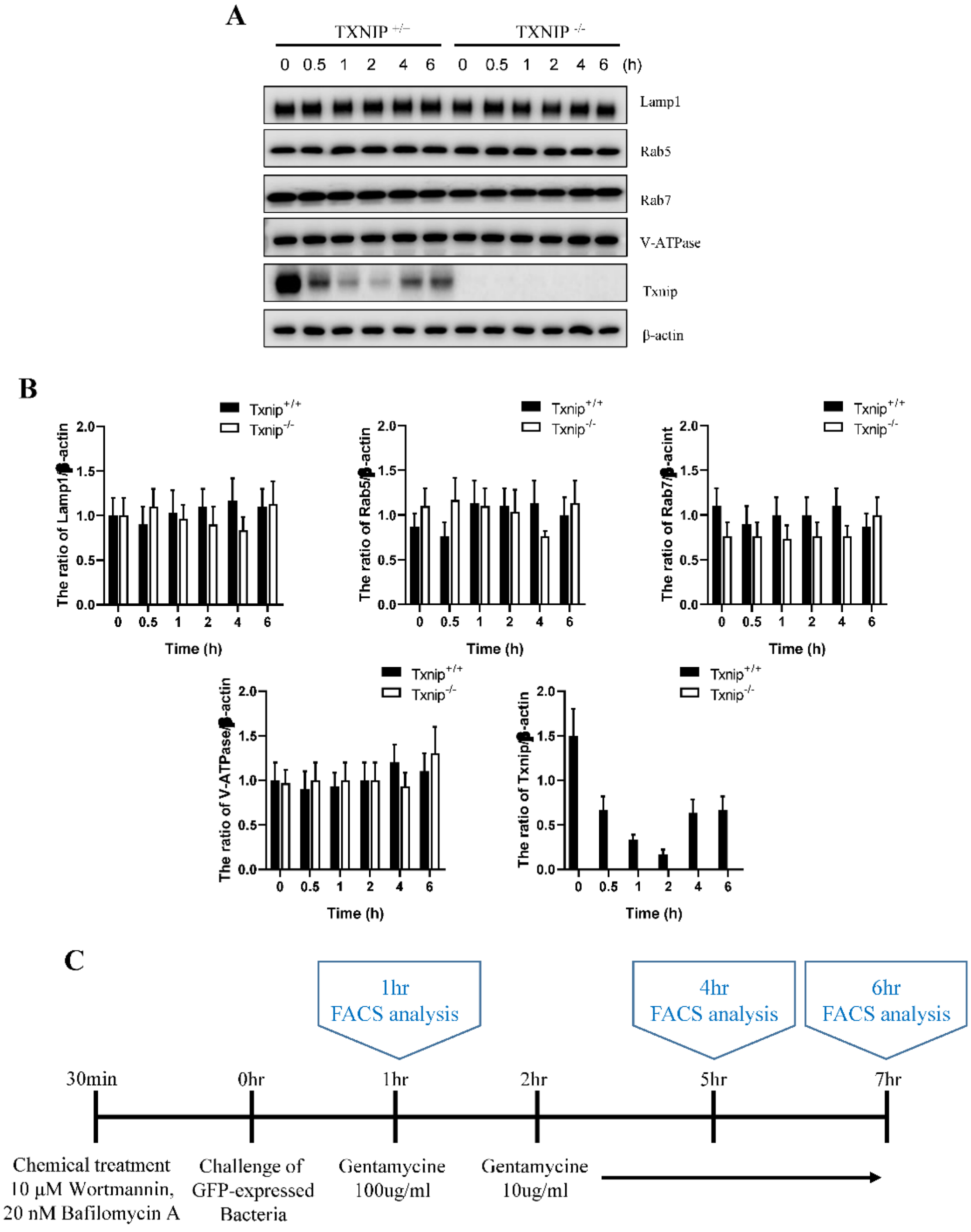

Supplementary Figure 3. (A) Expression of proteins related to phagosome maturation from WT and *Txnip* KO mouse peritoneal macrophages treated with *E. coli* for the indicated times. (B) The quantification data of Lamp1, Rab5, Rab7, V-ATPase, and Txnip based on western blotting data presented in (A). (C) The experimental scheme used for GFP-expressing *E. coli* treatment, removal of extracellular bacteria, and wortmannin or bafilomycin A treatment.

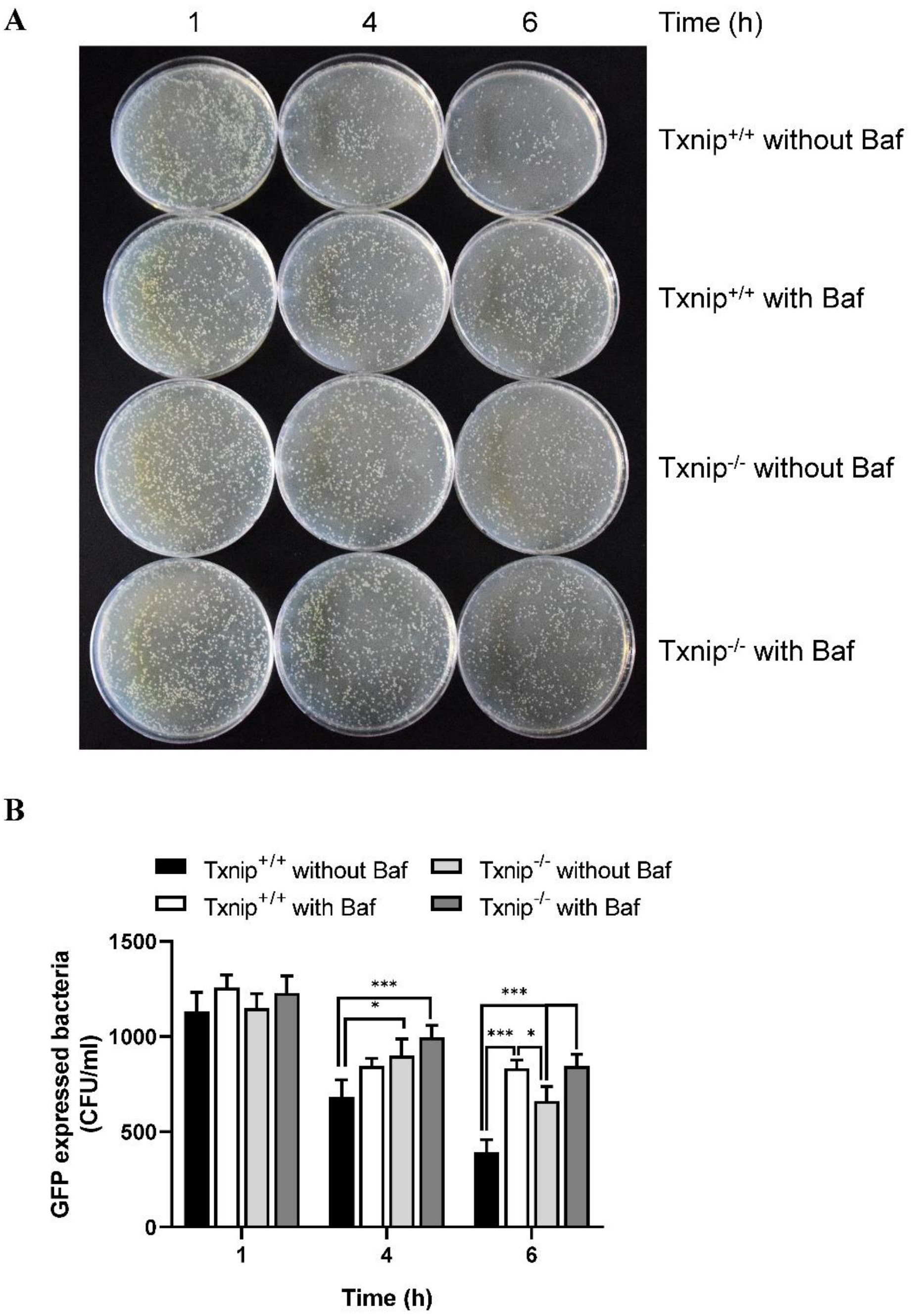

Supplementary Figure 4. (A) Representative LB agar plates after overnight incubation with cell extracts derived from WT and *Txnip* KO peritoneal macrophages incubated with GFP-expressing *E. coil* for the indicated periods. WT and *Txnip* KO peritoneal macrophages treated with bafilomycin A (20 nM) before incubation with GFP-expressing *E. coli*. (B) CFUs on LB agar plates after overnight incubation with cell extracts derived from WT and *Txnip* KO mouse peritoneal macrophages incubated with *E. coli* for the indicated times. Data are expressed as mean ± SD (n = 3, *P < 0.05, ***P < 0.001, compared with WT).

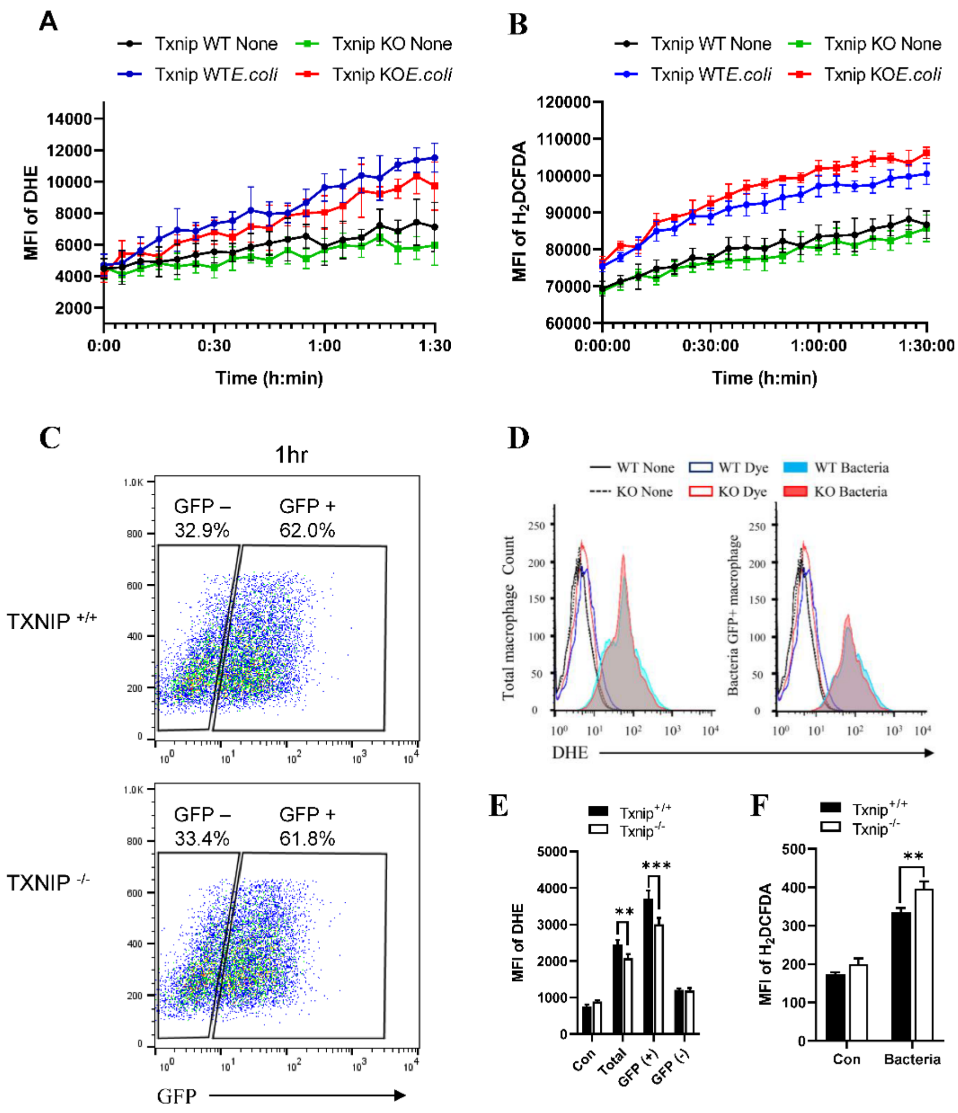

Supplementary Figure 5. (A) Mean fluorescence intensity (MFI) of DHE showing the ratio of superoxide level in WT and *Txnip* KO mouse peritoneal macrophages after treatment with *E. coli* at an MOI of 20 (n = 6). (B) The MFI of H_2DCFDA dye showing the ratio of superoxide level in WT and *Txnip* KO mouse peritoneal macrophages after treatment with *E. coli* at an MOI of 20 (n = 6). (C) FACS analysis showing the proportion of WT and *Txnip* KO mouse peritoneal macrophages, which retained GFP-expressing *E. coli* 1 h after treatment with GFP-expressing *E. coli* at an MOI of 20. (D) Distribution of WT and *Txnip* KO mouse peritoneal macrophages based on the intensity of DHE at 1 h after treatment with GFP-expressing *E. coli* at an MOI of 20. (E) The MFI of DHE in the total, GFP-positive, and GFP-negative WT and *Txnip* KO mouse peritoneal macrophages after treatment with GFP-expressing *E. coli* at an MOI of 20 for 1 h (n = 3). (F) The MFI of H_2DCFDA in WT and *Txnip* KO mouse peritoneal macrophages after treatment with *E. coli* at an MOI of 20 for 1 h (n = 3).

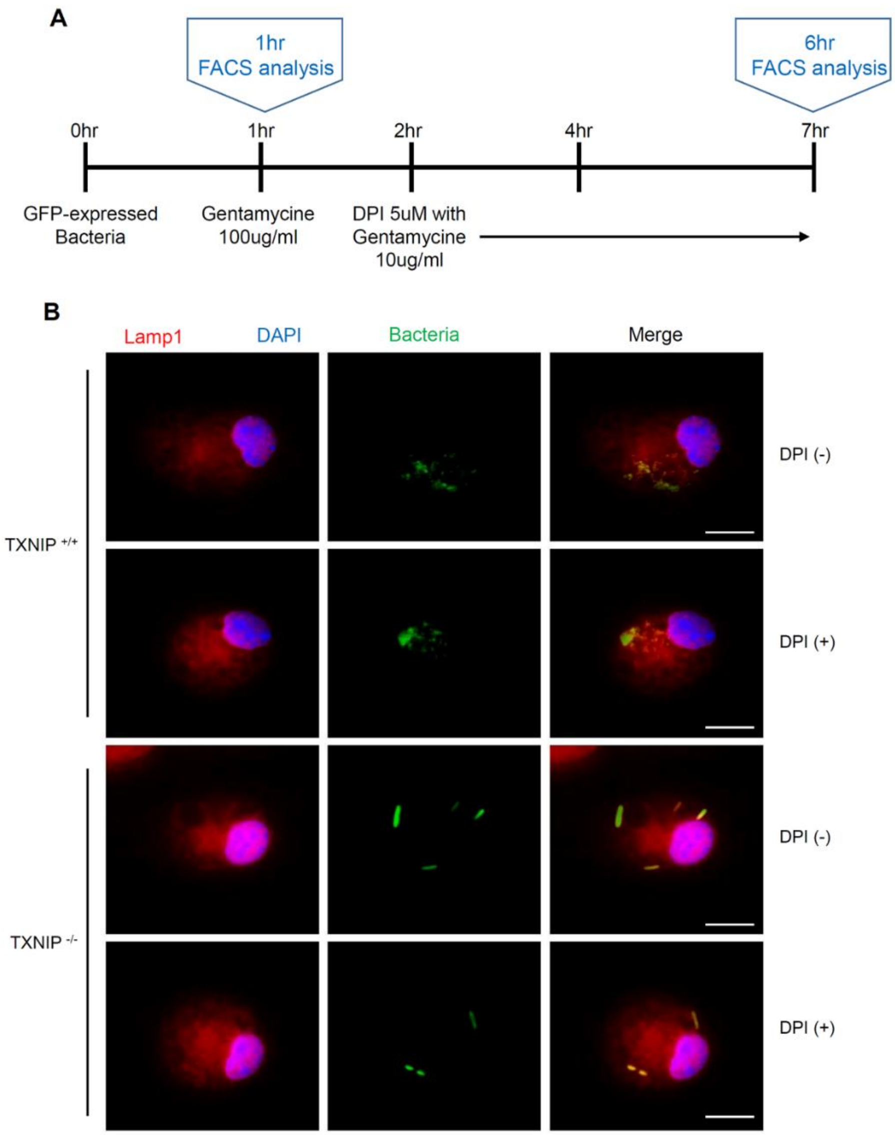

Supplementary Figure 6. (A) Experimental scheme used for GFP-expressing *E. coli* treatment, removal of extracellular bacteria, and DPI treatment. (B) Representative images of bacteria-laden WT and *Txnip* KO mouse macrophages 6 h after 1 h of treatment with GFP-expressing *E. coli*, with or without DPI treatment, and removal of extracellular bacteria. Scale bar, 10 µm.

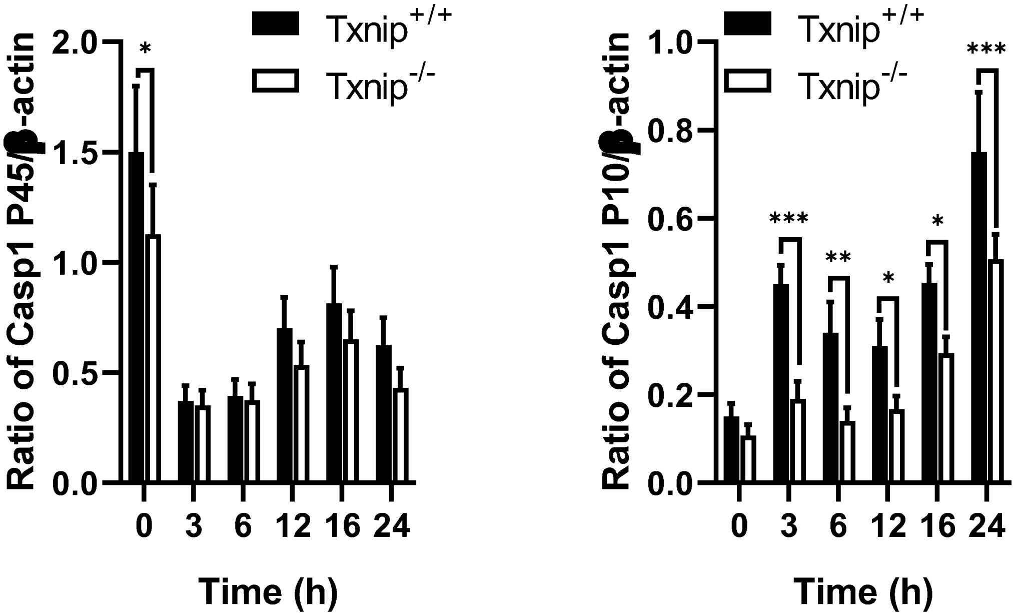

Supplementary Figure 7. Quantification of Casp1 P45 and Casp1 P10 using western blotting data shown in Figure 4B. Data are expressed as mean ± SD (n = 3, *P < 0.05, **P < 0.01, ***P < 0.001, compared with WT).
